# Retraction: Mineral biofortification of vegetables through soil-applied poultry mortality compost

**DOI:** 10.1371/journal.pone.0274207

**Published:** 2022-09-14

**Authors:** The PLOS ONE Editors

The *PLOS ONE* Editors retract this article [1] because it was identified as one of a series of submissions for which we have concerns about authorship, competing interests, and peer review. We regret that the issues were not addressed prior to the article’s publication.

All authors did not agree with the retraction.

## References

[1] Mubarak MU, Kiran A, Shahzad AN, Qayyum MF, Ishfaq M, Mahmood K, et al. (2022) Mineral biofortification of vegetables through soil-applied poultry mortality compost. PLoS ONE 17(2): e0262812. 10.1371/journal.pone.0262812 35113909 PMC8812912

